# Adequate Psychodrugs Do Not Impair Gait Speed in Older, Relatively Healthy, Independent Patients: A Cross-Sectional Study

**DOI:** 10.3390/healthcare14121706

**Published:** 2026-06-15

**Authors:** María Ángeles Caballero-Mora, Virginia Mazoteras-Muñoz, Irene Bartolomé-Martín, Luis Saucedo-Mora, Leocadio Rodríguez-Mañas, Ángel Rodríguez-Laso

**Affiliations:** 1 Geriatric Department, Hospital General Universitario de Ciudad Real, Línea de investigación TEC2022-002, Instituto de Investigación Sanitaria de Castilla la Mancha, IDISCAM, 13005 Ciudad Real, Spain; virginiamazoteras@gmail.com; 2 Departamento de Ciencias Médicas Básicas, Universidad San Pablo CEU, 28668 Madrid, Spain; ibarmar@hotmail.com; 3 Escuela Técnica Superior de Ingeniería de Aeronaútica y del Espacio, Universidad Politécnica de Madrid, 28040 Madrid, Spain; 4 Geriatric Department, Hospital General Universitario de Getafe, 28905 Getafe, Spain; leocadio.rodriguez@salud.madrid.org; 5 Unidad Docente Multiprofesional de Atención Familiar y Comunitaria Centro, Gerencia Asistencial de Atención Primaria, Consejería de Sanidad, Comunidad de Madrid, 28035 Madrid, Spain; arodriguezlaso@salud.madrid.org

**Keywords:** falls, gait speed, inadequate psychotropic agents, adequate psychotropic agents, psychotropic drugs, psychotropic medications, prescribing appropriateness, potentially inappropriate medications, older adults

## Abstract

**Highlights:**

**What are the main findings?**

- In the fully adjusted model, inappropriate psychotropic medication use (inadequate psychotropic agents) was observed to be associated with a 0.109 m/s slower gait speed compared with no use (B = −0.109), and this association was observed to be statistically significant (*p* = 0.026).
- In the fully adjusted model, compared with no use, adequate psychotropic medication use was not observed to be associated with gait speed (B = −0.018 m/s; *p* = 0.699).

**What is the implication of the main findings?**

- In our study, adequately prescribed psychotropic agents were not observed to be associated with gait speed. This finding is clinically relevant, as it suggests that continuing appropriately indicated psychotropic treatment may be feasible without an observed reduction in gait speed, a key risk factor for falls.

**Abstract:**

**Background/Objectives**: The relationship between psychotropic medication use, prescribing appropriateness, and fall-related risk factors remains incompletely characterised. Gait speed is a key predictor of falls. We aimed to examine whether gait speed is associated with appropriately versus inappropriately prescribed psychotropic medication use among relatively healthy older adults. **Methods**: We conducted an observational cross-sectional study of 119 community-dwelling adults aged ≥ 70 years with low comorbidity burden (Charlson Comorbidity Index < 2) and preserved functional status (Barthel Index > 85). Gait speed was assessed over 6 metres. Psychotropic medication use was recorded and prescribing appropriateness was evaluated using STOPP/START and Beers criteria, supplemented by geriatric pharmacological considerations. Multivariable linear regression analyses adjusted for age, sex, waist-to-height ratio, and frailty status. **Results**: In the fully adjusted model, inappropriate psychotropic medication use was associated with significantly slower gait speed compared with no use (B = −0.109 m/s; *p* = 0.026). In contrast, appropriately prescribed psychotropic medication use was not associated with gait speed (B = −0.018 m/s; *p* = 0.699). **Conclusions**: In this cross-sectional sample of relatively healthy older adults, appropriate psychotropic medication use was not associated with gait speed impairment, whereas inappropriate use was associated with slower gait. Although causal inference is not supported, these findings may inform prescribing quality and fall-risk assessment in older populations.

## 1. Introduction

The WHO considers that falls are a major public health problem affecting at least one-third of people aged 70 and older [1]. They produce a great impact on older people, because they cause higher mortality, disability, sanitary costs, hospitalisation and death. For this reason, it is important to prevent falls before they take place, identifying and acting on several of the risk factors for falls. Among these risk factors, gait speed has been identified as one of the most important ones [2,3,4,5,6].

In this same regard, randomised controlled trials of fall prevention have shown the effectiveness of several types of interventions, like the reduction of body mass index (BMI), frailty and polypharmacy, including the withdrawal of drugs like antihypertensives, diuretics, and antiarrhythmic and psychotropic agents [7,8,9,10,11,12]. While introducing changes in the other groups of drugs is usually suitable, in clinical practice in this population retiring/substituting psychotropic agents is problematic. The reason for this is the high prevalence of depression, insomnia and anxiety that does not always make their withdrawal possible [13]. To be able to maintain the prescription of this useful drug, it would be very relevant to know if adequate psychotropic agents according to the STOPP/START [14] and BEERS [15] criteria increase the risk for falls or at least one of its main predictors, a slower gait speed.

Previous studies have examined the association between psychotropic medication use and gait; however, most do not distinguish between appropriate and inappropriate prescribing, thereby limiting the clinical interpretability of their findings [16,17,18].

Our study goal is to analyse whether there is a different association between adequate and inadequate psychotropic agents and gait speed in people older than 70 years with a relatively preserved health status.

## 2. Materials and Methods

### 2.1. Setting and Study Design

We designed an observational cross-sectional pilot study. The study was conducted from January 2016 to November 2018 at the outpatient Geriatric Medicine clinic of Hospital General Universitario de Getafe, Getafe, Spain, using a cross-sectional design with single-visit assessments. All clinical assessments were performed by a consultant physician specialising in Geriatric Medicine. Inclusion criteria were being 70 years or older, living in the community and being able to walk without aids with a normal gait (this was subjectively determined by an expert explorer). Patients with a Mini-Mental State Exam < 25 [19]; Barthel < 85 [20]; terminal illness (life expectancy < 6 months); unwillingness to participate; acute medical illness in the past 6 months; major depression; neurological diseases such as Parkinson’s disease, cerebellar disease, and peripheral neuropathy; and peripheral artery and coronary heart disease were excluded.

### 2.2. Measures of Psychotropic Drugs

We applied the ATC classification [21] and considered subgroups N05 and N06 and the doses taken during at least six months without modifications. The classification of the drugs as “adequate” vs. “non-adequate” was done following the STOPP-START [14] and Beers [15] criteria, but also considerations on prolonged half-life in older people and the existence of active metabolites or adverse effects like strong hypnotic, sedative or anticholinergic effects [22]. If a participant was taking both adequate and inadequate psychotropic drugs, they were considered to be receiving inadequate drugs. According to the previously cited criteria, individuals taking 10 mg Zolpidem per day (N = 3), 1–1.5 mg Bromazepam per day (N = 5), 0.25 mg Alprazolam per day (N = 1), 1 mg Lormetazepam per day (N = 3), 7.5 mg Midazolam per day (N = 1), 5 mg Diazepam per day (N = 2), 5 mg Clorazepate Dipotassium per day (N = 1), 0.5 mg Clonazepam per day (N = 1), 20–30 mg Fluoxetine per day (N = 2), 20 mg Paroxetine per day (N = 5) (these last two selective serotonin reuptake inhibitors because of their prolonged half-life in the case of Fluoxetine or strong anticholinergic effects in the case of Paroxetine), and 25 mg Amitriptyline per day (N = 1) were considered to be receiving an inadequate treatment due to prolonged half-life in older people and the existence of active metabolites or adverse effects like strong hypnotic, sedative or anticholinergic effects. Individuals taking 0.5–1 mg Lorazepam (a benzodiazepine with a short half-life without active metabolites) per day (N = 25, of which 4 were considered inadequate because they were taking Paroxetine, Fluoxetine or Lormetazepam in addition), 10 mg Escitalopram per day (N = 1), 10–20 mg Citalopram per day (N = 4, of which 1 was considered inadequate because they were taking Bromazepam in addition), 15 mg Vortioxetine per day (N = 1), 50–100 mg Sertraline per day (N = 8, of which 3 were considered inadequate because they were taking Zolpidem, Clonazepam or Alprazolam in addition), 50–100 mg Trazodone per day (N = 2), 15–30 mg Mirtazapine per day (N = 4), 50 mg Venlafaxine per day (N = 1), and 30 mg Duloxetine per day (N = 1) were considered to be receiving an adequate treatment. One participant taking four psychotropic drugs was classed as adequate. Of 4 participants taking three psychotropic drugs, two were in the adequate group and two in inadequate. Of 11 participants taking two psychotropic drugs, six were in the adequate group and five in inadequate.

### 2.3. Descriptive Factors

In order to describe the sample, we recorded sociodemographic characteristics (age, gender), basic activities of daily living—BADL (Barthel Index [20]) and instrumental activities of daily living—IADL (Lawton and Brody Index [23]), cognition (MMSE [19]), comorbidity (Charlson Index [24]), BMI (body mass index) and number of falls in the past year.

Basic activities of daily living were assessed using Barthel Index, a 0–100 scale that evaluates independence in basic activities of daily living (e.g., feeding, bathing, dressing and mobility), with higher scores indicating greater independence.

Instrumental activities of daily living were assessed using the Lawton (IADL) Index, which captures more complex functions necessary for independent community living (e.g., using the telephone, shopping, food preparation, housekeeping, laundry, transportation, medication management and finances), with higher scores indicating greater independence.

Comorbidity burden was quantified using the Charlson Comorbidity Index, a weighted summary score of chronic conditions that is widely used to estimate overall comorbidity severity and prognosis, with higher scores indicating greater comorbidity.

Global cognitive function was assessed using the Mini-Mental State Examination (MMSE), a brief, widely used screening tool of cognitive status (score range 0–35) that evaluates orientation, registration, attention and calculation, recall, language and visuospatial abilities. Higher scores indicate better cognitive performance, and the MMSE is commonly used in clinical and research settings to characterise cognitive impairment and to support eligibility or stratification decisions.

### 2.4. Gait Speed

Gait speed, as assessed using the 6 m test, was the outcome variable. The subjects walked a total of 10 m at their usual pace. The stopwatch was started after having walked 2 m and stopped when the individual reached the 8 m mark. We took two measures and chose the best in metres per second (m/s).

### 2.5. Confounding Factors

The following variables were used as confounders, following the same criteria of other authors [25]: age, gender, waist-to-height ratio below or above 0.65 and number of Fried frailty criteria standardised to the Spanish population [26]. We excluded number of falls because it could be an adverse effect of psychotropic drugs.

### 2.6. Statistical Analyses

Variables were described using arithmetic means and standard deviations and proportions where appropriate. Bivariate analyses with Student’s *t*-test and ANOVA were carried out. Before hypothesis testing, we assessed model assumptions: normality of distributions (Shapiro–Wilk or Kolmogorov–Smirnov, as appropriate), homogeneity of variances (Levene’s test), linearity (scatterplot matrices and residual plots) and independence (Durbin–Watson). Post hoc pairwise comparisons were not performed, as the ANOVA was used as an omnibus test to assess overall between-group differences and the study was not designed to evaluate specific pairwise contrasts.

Linear regression was used to analyse the association between gait speed and the other variables (age, gender, waist-to-height ratio, number of Fried criteria, and consumption of psychotropic drugs, split into none, adequate and inadequate). We checked regression assumptions, specifically the normality and homoscedasticity of residuals because of the non-normal distribution of gait speed.

All statistical tests were performed at the 0.05 level. Statistical tests were performed using SPSS for Windows (version 13.0; SPSS Inc., Chicago, IL, USA).

### 2.7. Sample Size

The sample size for this study was determined a priori for a multiple linear regression model examining the association between psychotropic medication use (primary exposure with three categories: appropriate use, no use, and inappropriate use) and gait speed (continuous outcome), adjusted for age, sex, frailty, and waist-to-height ratio (7 parameters in total). Monte Carlo simulations by Austin and Steyerberg suggest that linear regression models may require as few as 2 subjects per variable to obtain regression coefficients with <10% relative bias; however, to further limit overfitting and ensure precise estimates, we also applied Green’s rule-of-thumb for etiological models (N ≥ 50 + 8 predictors, i.e., ≥106 participants for number of predictors = 7) and the recommendations of Riley et al. advocating approximately 15–20 participants per parameter (≈105–140 for our model) [27,28,29,30]. Accordingly, we aimed to recruit 100–120 participants to achieve adequate power (≈80–90% at α = 0.05) and robust estimates with sufficient representation across exposure categories; the final analysed sample comprised 119 participants.

## 3. Results

### 3.1. Sample Characteristics (Table 1)

A total of 235 subjects met the inclusion criteria. Of the 235 individuals who met the inclusion criteria and were informed about the study, 17 declined to participate. Of the 218 individuals who agreed to take part, 91 were excluded according to the prespecified exclusion criteria (11 due to limiting osteoarthritis, 8 due to parkinsonism or Parkinson’s disease, 9 due to cerebrovascular disease, 24 due to dementia or the MEC-based exclusion criterion, 10 due to major depression or follow-up in Mental Health services, 5 due to peripheral artery disease or peripheral neuropathy, 9 due to ischemic heart disease, 2 due to severe visual impairment, 9 due to episodes of vertigo in the previous 6 months, and 4 due to active cancer with poor prognosis). Of the remaining 127 individuals, 4 did not attend the examination visit. A total of 123 subjects therefore underwent the baseline assessment. Three were subsequently excluded from the analytical sample (one because of an episode of vertigo during the assessment and two because they had an ankle–brachial index below 0.9). In addition, Fried frailty criteria were not available for one participant, who was therefore excluded. The final analysable baseline sample consisted of 119 participants. Out of those, 46 consumed psychotropic drugs; 22 were considered to take them inadequately and 24 adequately. Mean age was 77 years (range 70–96 years), and 71.4% were women.

Our sample was free of significant disability and comorbidity. The Barthel Index average was 99, Lawton Index average was 7 and Charlson Index average was less than 1. Regarding cognition, the MMSE average was 31. Subjects had a BMI average of 29. Regarding the frailty status, 40 (33.7%) subjects were frail or prefrail, while 75 (63%) were robust. When we analysed each criterion of frailty (with respect to our total sample), 13 (10.9%) showed involuntary weight loss, 18 (15.1%) weakness, 1 (0.8%) slowness, 15 (12.6%) exhaustion and 15 (12.6%) low activity and 20 (16.8%) suffered from polypharmacy.

**Table 1:** Descriptive variables (N = 119 subjects).

Variable	Mean	SD
Age	77.7	5.8
Barthel Index	99.0	2.1
Lawton Index	7.4	1.2
Mini-Mental State Examination Score	31.7	3.3
Charlson Index	0.5	0.8
Body Mass Index (kg/m^2^)	28.8	4.0
Number of Falls	0.6	1.1
Gait Speed (m/seg)	1.1	0.3
**Variable**	**N**	**Ratio**
Women Frailty	85	71.4%
Frail	4	3.4%
Prefrail	37	30.8%
Robust	78	65%
Weight Loss	14	11.7%
Weakness	23	19.2%
Slowness	1	0.8%
Exhaustion	8	6.7%
Low Activity	15	12.6%
Inadequate Use of Psychotropic Agents	22	18.5%
Adequate Use of Psychotropic Agents	24	20.2%
Polypharmacy (6 or more)	20	16.8%

Characteristics of the sample tested by variables. SD = standard deviation. N = number of cases.

### 3.2. Univariate and Bivariate Analyses of Gait Speed (Table 2)

The mean gait speed at six metres was 1.14 m/s (sd 0.27; Table 1). All bivariate analyses showed a significant association with gait speed. Those who did not consume psychotropic agents walked at 1.19 m/s, while those consuming adequate ones walked at 1.08 m/s and those consuming inadequate ones at 1.01 m/s (*p* = 0.013). The octogenarians and nonagenarians (0.97 m/s ± 0.26) walked more slowly than septuagenarians (1.23 m/s ± 0.23; *p* < 0.001). Men walked faster (1.28 m/s ± 0.27) than women (1.08 m/s ± 0.25; *p* < 0.001). The group with a waist-to-height ratio less than 0.65 walked faster (1.17 m/s ± 0.25) than those with a waist-to-height ratio equal or greater than 0.65 (1.06 m/s ± 0.30; *p* = 0.034). Analysing frailty, the four frail subjects showed a slower gait speed (0.79 m/s ± 0.06) compared to prefrail/robust subjects (1.14 m/s ± 0.25). These results are presented in Table 2.

**Table 2:** Bivariate analyses of gait speed.

Groups (N = 119)	Gait Speed 6 m (m/s) Mean ± SD (95% CI)	*p*
Age		*p* < 0.001
70–79 years (N = 76)	1.23 ± 0.23 (1.18–1.28)	
80 years and >80	0.97 ± 0.26 (0.89–1.05)	
Gender		*p* < 0.001
Men (N = 34)	1.28 ± 0.27 (1.19–1.38)	
Women (N = 85)	1.08 ± 0.25 (1.02–1.13)	
Waist-to-height ratio (cm/cm)		*p* = 0.034
<0.65 (N = 79)	1.17 ± 0.25 (1.12–1.23)	
≥0.65 (N = 40)	1.06 ± 0.30 (0.96–1.15)	
Frail		*p* = 0.008
Robust or prefrail/frail (N = 115)	1.15 ± 0.26 (1.10–1.20)	
Frail (N = 4)	0.69 ± 0.25 (0.29–1.09)	
Psychotropic drugs		*p* = 0.013
Inadequate (N = 22)	1.01 ± 0.26 (0.89–1.12)	
Adequate (N = 24)	1.08 ± 0.28 (0.96–1.20)	
No psychotropic agents (N = 73)	1.19 ± 0.26 (1.12–1.25)	

Stratification of the sample into groups according to variables that may influence gait. Bivariate analyses with Student’s *t*-test and ANOVA were carried out. N = sample size; 95% CI = 95% confidence interval; Mean ± SD = mean ± standard deviation; *p* = *p*-value.

### 3.3. Multivariate Model (Table 3)

When we carried out a linear multivariate analysis (Table 3), we obtained similar results to bivariate analyses for age and gender. Per each additional year of age, subjects walked 0.021 m/s slower (*p* < 0.001) and women walked 0.183 m/s slower than men (*p* < 0.001). Adjustment introduced important changes in the rest of the variables. In the adjusted model, per each additional 0.1 waist-to-height ratio, subjects walked 0.838 m/s slower (*p* = 0.004). Per each frailty criterion the subjects walked 0.088 m/s slower (*p* = 0.007). Subjects who took inadequate psychotropic agents walked 0.109 m/s slower than those who did not take psychotropic drugs (*p* = 0.026), while those who took adequate psychotropic agents did not show any relevant difference with those who did not take psychotropic agents (−0.018 m/s, *p* = 0.699) after adjustment.

**Table 3:** Multivariate analysis model (linear regression).

N = 119	Bivariate Model	Multivariate Model R^2^ 0.456
**Age (years)**	**B = −0.022**	**B = −0.021**
	***p*** **< 0.001**	***p*** **< 0.001**
	**R^2^ = 0.233**	
**Gender (men)**	**B = 0.208**	**B = 0.183**
	***p*** **< 0.001**	***p*** **< 0.001**
	**R^2^ = 0.118**	
**Waist-to-height ratio (cm/cm)**	**B = −1.261**	**B = −0.838**
	***p*** **< 0.001**	***p*** **= 0.004**
	**R^2^ = 0.093**	
**Frail** (number of criteria)	**B = −0.209**	B = **−0.088**
	***p*** **< 0.001**	***p*** **= 0.007**
	**R^2^ = 0.236**	
**Inadequate psychotropic drugs**	**B = −0.154**	**B = −0.109**
	***p*** **= 0.016**	***p*** **= 0.026**
	**R^2^ = 0.048**	
**Adequate psychotropic drugs**	B = −0.066	B = −0.018
	*p* = 0.291	*p* = 0.699
	R^2^ = 0.009	

Linear regression analyses of factors potentially influencing gait. The bivariate model column presents the results of separate linear regression for each explanatory variable in relation to gait speed. The multivariable model column presents the association between gait speed and all prespecified covariates entered simultaneously in the model: age, sex, waist-to-height ratio, number of Fried frailty criteria, inadequate psychotropic drugs and adequate psychotropic drugs. N = sample size; B = (unstandardised regression coefficient); R^2^: R-squared, coefficient of determination; *p* = *p*-value.

## 4. Discussion

Although the overall sample size and subgroup sizes may appear relatively small, the study sample was determined a priori based on the requirements of the primary analytical model. The sample size calculation was performed for a multiple linear regression framework examining the association between psychotropic medication use and gait speed, adjusted for key covariates. Following established recommendations for multivariable models, the target sample size was set to ensure an adequate number of participants per parameter, minimising the risk of overfitting and providing sufficient statistical power to detect clinically meaningful associations. The final analysed sample met these predefined criteria and was therefore considered appropriate for addressing the primary study hypothesis, while recognising that subgroup analyses may remain underpowered and should be interpreted with caution.

In this cross-sectional study of relatively healthy community-dwelling older adults, inappropriate psychotropic medication use was observed to be associated with slower gait speed, whereas appropriately prescribed psychotropic medications were not associated with gait speed. The magnitude of the observed association for inappropriate use exceeded one-tenth of a metre per second, which is commonly regarded as the smallest clinically meaningful difference in gait speed [31].

Several previous studies have reported an association between psychotropic medication use and falls [7,8,9,10,32,33,34]; however, fewer have specifically examined gait speed, and most have not distinguished between appropriate and inappropriate prescribing [3,6,16,17,18]. Moreover, many studies do not report specific drugs or doses, despite the well-recognised dose-dependent effects and adverse profiles of psychotropic medications in older adults. In this context, the absence of an observed association between gait speed and appropriately prescribed psychotropic medications in our study should be interpreted cautiously and not as evidence of absence of effect. For example, in the case of Lorazepam we have considered its consumption adequate following the STOPP/START and BEERS criteria given that all subjects in our study were taking the drug at very low doses, the highest dose being 1 mg per day. The fact that we found no association with walking speed at these doses does not mean that the drug may not be associated at higher doses nor that we recommend the drug either, because there is evidence that benzodiazepines can have other side effects in the elderly, such as cognitive impairment [32,33,34]. More studies with a larger sample analysing drug by drug would be necessary to be able to draw more detailed conclusions, but this work does open up an interesting line of research, since it seems that not all psychotropic drugs work the same in relation to gait speed according to their characteristics and doses and therefore should not be considered as a single group.

A key feature of the present analysis is that the findings were obtained in a sample with preserved functional status (free from disability, functional impairment, cognitive deterioration and repeated falls), low comorbidity burden, and a mean gait speed (1.14 m/s) above the commonly accepted threshold of 1.0 m/s. This suggests that clinically relevant differences in gait speed may be detectable before overt functional decline or recurrent falls occur. From a preventive perspective, this is particularly relevant, given the substantial individual and societal consequences associated with falls in older populations [3,4]. Our results show that in our study the use of adequate psychotropic agents does not change gait speed. However, further studies are needed to confirm this hypothesis; this raises the opportunity of substituting adequate drugs for inadequate ones in people at risk for falls when the withdrawal of drugs is impossible in cases of depression, insomnia and anxiety, which are very prevalent conditions in this population [13].

### Limitations

The observational nature of our study prevents us from proving that switching inadequate prescription of psychotropic agents to an adequate one or no prescription would improve gait speed. In addition, its cross-sectional nature does not allow to establish that the inadequate prescription was followed by an impairment of speed.

Another limitation of the study is that different classes of psychotropic medications were grouped together despite their heterogeneous pharmacological profiles. This approach was chosen a priori to address the primary research question, which focused on prescribing appropriateness rather than on the effects of individual drug classes, and to preserve statistical power given the relatively small sample size. Stratified analyses by drug class were therefore not feasible, and potential class-specific associations may have been diluted and cannot be excluded. Residual confounding should also be considered when interpreting these findings. The analyses were not adjusted for specific depression, anxiety, or insomnia scales due to time constraints during the assessment. Although individuals with major depression according to DSM-IV criteria were excluded, and the associations persisted after adjustment for frailty (which includes exhaustion, reduced activity, and weight loss), confounding by indication cannot be fully ruled out. In addition, all psychotropic medications had been taken at stable therapeutic doses for at least six months prior to assessment, reducing the likelihood of acute treatment effects, but not excluding the influence of subclinical affective symptoms or differences in indication severity.

The overall good health of our sample limits the external validity of our study for populations in worse health. As a result, the analysable sample should be considered representative of community-dwelling older adults who are medically stable and able to complete the assessment protocol, rather than of the broader older population that includes individuals with advanced multimorbidity, significant cognitive impairment, neurological disorders, severe musculoskeletal limitation, or relevant cardiovascular/vascular disease.

Finally, the cross-sectional design of the study precludes causal inference. The observed associations should therefore be interpreted as descriptive and hypothesis-generating. Longitudinal studies with larger samples, systematic assessment of psychiatric symptoms, and analyses by pharmacological class and dose are needed to better disentangle the effects of psychotropic medications from those of their underlying indications.

## 5. Conclusions

This study reinforces the importance of appropriate prescribing in older adults, highlighting that in our study adequately prescribed psychotropic medication use was not associated with gait speed impairment, a key determinant of fall risk.

This is important because when talking about the withdrawal of psychotropic agents as an effective intervention to prevent falls, no distinction is made between inadequate and adequate psychotropic agents, although there is scientific evidence that not all drugs are the same when it comes to treating older subjects. Further studies should be carried out to see if substituting adequate for inadequate drugs increases gait speed.

## Data Availability

The data are not available as they belong to a private database.

## Abbreviations

The following abbreviations are used in this manuscript:

ATC	Anatomical Therapeutic Chemical (classification system)
B	Unstandardised regression coefficient
BADL	Basic activities of daily living
BMI	Body mass index
CIBERFES	Instituto de Salud Carlos III (Spain)
IADL	Instrumental activities of daily living
ISCIII	Biomedical Research Networking Centre on Frailty and Healthy Ageing (Spain)
mg	Milligram
MMSE	Mini-Mental State Examination
m/s	Metres per second
N	Sample
*p*	*p*-value
R^2^	R-squared, coefficient of determination
SD	Standard deviation
WHO	World Health Organization
95% CI	Confidence interval
α	Significance level (Type I error rate)
